# HLA Allele Prevalence in Disease-Modifying Antirheumatic Drugs-Responsive Enthesitis and/or Arthritis Not Fulfilling ASAS Criteria: Comparison with Psoriatic and Undifferentiated Spondyloarthritis

**DOI:** 10.3390/jcm10143006

**Published:** 2021-07-06

**Authors:** Elvira Favoino, Livio Urso, Alessandra Serafino, Francesca Misceo, Giacomo Catacchio, Marcella Prete, Federico Perosa

**Affiliations:** Department of Biomedical Science and Human Oncology (DIMO), Rheumatic and Systemic Autoimmune Diseases Unit, University of Bari Medical School, Piazza G. Cesare 11, I-70124 Bari, Italy; elvira.favoino@uniba.it (E.F.); livio.urso@gmail.com (L.U.); aleserafik@gmail.co (A.S.); franc.misceo@gmail.com (F.M.); giaco.catacchio@gmail.com (G.C.); marcella.prete@uniba.it (M.P.)

**Keywords:** spondyloarthritis, human leukocyte antigen, undifferentiated enthesitis and/or arthritis, ASAS classification criteria, clinical management

## Abstract

Spondyloarthritis (SpA) is a group of inflammatory rheumatic diseases characterized by common clinical features, such as inflammatory enthesitis, arthritis and/or back pain. SpA is strongly associated with human leukocyte antigen (HLA) class I allotype B27. Ankylosing spondylitis has historically been the SpA subgroup with one of the strongest, best-proven associations with HLA-B27. The remaining SpA subgroups, namely psoriatic arthritis (PsA), inflammatory bowel diseases-associated arthritis/spondylitis, reactive arthritis, and undifferentiated SpA (uSpA), have also been associated with HLA allotypes other than HLA-B27. In this retrospective study, we analyzed the association between the HLA class I and II haplotypes and the susceptibility to enthesitis and/or arthritis (E/A). Special attention was paid to E/A responding to disease-modifying antirheumatic drugs (DMARDs) not fulfilling ASAS classification criteria (ASAS^−^), as compared to ASAS^+^ forms including PsA and uSpA. The whole E/A group showed significant independent associations with HLA-A28(68), B27, Cw3, Cw12, and DQ1; taken singly, PsA was associated with HLA-B27 and DQ1, uSpA with HLA-B16(38,39) and B27, and E/A ASAS^−^ with HLA-A28(68), Cw8, and Cw12. This study identified novel risk HLA allotypes for different SpA subgroups in an Italian population. HLA typing could aid the diagnosis and treatment of E/A subgroups, including DMARDS-responsive forms not fulfilling ASAS classification criteria.

## 1. Introduction

Spondyloarthritis (SpA) is a heterogeneous group of chronic inflammatory rheumatic diseases encompassing different clinical subgroups, namely ankylosing spondylitis (AS), psoriatic arthritis (PsA), inflammatory bowel diseases-associated arthritis/spondylitis (enteropathic arthritis), reactive arthritis, and undifferentiated SpA (uSpA). The latter includes patients with enthesitis and/or arthritis (E/A) fulfilling ASAS classification criteria [1] (E/A ASAS^+^) but who cannot be included under the classification criteria of any of the single well-defined ASAS subgroups. SpA can also be clinically classified as axial, peripheral, or combined forms, depending on whether axial or peripheral joints or both [1,2,3] are the predominant sites affected by clinical manifestations.

SpA and its subgroups share common clinical manifestations, namely enthesitis, inflammatory back pain, sacroiliitis, peripheral arthritis, uveitis, and/or gut inflammation [4,5]. Among these, enthesitis is the most peculiar to SpA [6].

In daily practice, the rheumatologist is very often faced with patients showing signs and symptoms consistent with E/A. Even in the presence of ultrasound-documented E/A, the diagnosis of SpA is not always straightforward when 2011 ASAS criteria are not fulfilled [1] (i.e., patients with only E/A; E/A ASAS^−^), ASAS^+^ patients’ disease features do not satisfy any of the classification criteria related to SpA subgroups (uSpA), and/or patient-reported symptoms are not clearly inflammatory [7]. In these cases, the possibility of a mechanical/metabolic E/A cannot be ruled out [6]. Even so, a subgroup of E/A ASAS^−^ patients can still respond efficiently to DMARDs. Due to this uncertainty, it would be helpful to rely on markers to (a) better define E/A ASAS^−^ patients and/or (b) predict a good response to DMARDs and eventually to more advanced therapies [8].

The etiology of SpA is unknown, although it is believed to be multifactorial, with a major genetic predisposition, mostly consisting of the presence of human leukocyte antigen (HLA) alleles such as HLA-B27 [9]. The HLA-B27 prevalence varies markedly according to the SpA subtype and ethnicity [10,11], ranging from about 15% to 20% in PsA patients to over 95% in AS patients [12]. The same applies to uSpA, although in this case the prevalence of HLA-B27 ranges from 25% to 70% [13]. Besides HLA-B27, other major histocompatibility complex (MHC) alleles have been implicated in SpA susceptibility, including HLA-B8 [14], HLA-B15 [15,16,17], HLA-B16 with its splits, namely HLA-B38 and -B39 [14,18], HLA-DR1 [15,17] and DR4 [17].

The aim of this study was to investigate the HLA profile in a Caucasian cohort of patients affected by E/A, paying particular attention to the ASAS^−^ as compared to the ASAS^+^ subgroups, namely PsA and uSpA.

## 2. Patients and Methods

From 2013 to 2018, 113 consecutive patients with symptomatic peripheral E/A confirmed through ultrasound were recruited at the Rheumatology Research program Unit of the University of Bari. Patients with rheumatoid arthritis (fulfilling the 2010 ACR/EULAR classification criteria), as well as patients with AS, enteropathic, or reactive arthritis, were excluded. Patients with PsA were defined according to CASPAR criteria [19], while uSpA were defined according to the ASAS criteria for peripheral SpA in the absence of a more definite diagnosis (E/A ASAS^+^). Patients with E/A, responding to DMARDs, but not fulfilling ASAS criteria, were defined as E/A ASAS^−^. For each patient, data related to gender, age, and age at the onset of first symptoms, and medical history, including the presence of cardiovascular risk, metabolic syndrome, other autoimmune diseases (systemic or organ specific), fibromyalgia and malignancies, were recorded. Response to DMARDs was assessed according to DAPSA criteria, considering a minimum improvement of 50% from baseline [20,21]. Controls included a total of 318 HLA-I and -II serotyped healthy donors (HD) (female to male ratio 1:1). The study was approved by the Ethics Committees of the University of Bari. All participants gave written informed consent to enrollment as part of a project to study HLA disease markers.

## 3. Statistical Analysis

Statistical analyses were performed using Statistical Package for Social Sciences (SPSS) software (v21 for Windows). The Mann–Whitney *U* test was used to compare the differences between groups for continuous variables. Comparisons between groups for nominal variables were performed using Fisher’s exact test. Variables with statistically significant associations were analyzed by multivariate logistic regression, adjusting for gender as confounding variable. Multivariable logistic regression was performed to define the independent association between variables and SpA. For all tests, a *p*-value < 0.05 was taken to indicate statistical significance.

## 4. Results

In this retrospective study, 113 patients with E/A were enrolled, of which 73 ASAS^+^ (54 patients with PsA, 19 patients with uSpA), and 40 ASAS^−^, responsive to conventional DMARDS. The clinical characteristics of the study population are described in Table 1. The female to male ratio in the whole E/A cohort (wE/A) was 4:1, mean age ± SD was 53.8 ± 11.4, and mean age at the onset of the first symptoms was 44.5 ± 12.7. The mean body mass index (BMI) was 26.7 ± 4.4. No individuals in the uSpA or E/A ASAS^−^ groups had cutaneous psoriasis (Table 1). In addition, the HD group female to male ratio was 1:1 with a mean age ± SD of 34.82 ± 12.21.

As expected, the item “family history of psoriasis” was statistically higher in the PsA than in the uSpA (*p* < 0.001) or ASAS^−^ (*p* < 0.001) subgroups (Table 2). Variables such as “peripheral and axial arthritis,” “smoking,” “hypertension,” “cardiovascular risk,” “metabolic syndrome,” “autoimmune thyroiditis,” “fibromyalgia,” and “malignancy” were similarly distributed between subgroups (Table 2). Interestingly, significant differences in age at the onset of symptoms were found between PsA and ASAS^−^ (*p* = 0.03), being higher in ASAS^−^. As expected, the HLA-B27 distribution percentage in the wE/A group (*p* = 0.001) and its subgroups, PsA (*p* = 0.001) and uSpA (*p* < 0.001), was significantly higher than in the HD group (Table 3).

Fisher’s exact test was performed to define the association between HLA allotypes and wE/A or its subgroups (Table 4). wE/A was directly associated with HLA-A28(68) (*p* = 0.001), B27 (*p* = 0.001), Cw3 (*p* = 0.022), Cw8 (*p* = 0.023), Cw12 (*p* = 0.004), DQ1 (*p* = 0.005), and DQ3 (*p* = 0.038), and inversely with Cw7 (*p* = 0.004) and DR3 (*p* = 0.008).

PsA was directly associated with HLA-B17(57.58) (*p* = 0.035), B27 (*p* = 0.001), DQ1 (*p* = 0.036), and DQ3 (*p* = 0.030), whereas Cw7 (*p* = 0.016) and DQ7 (*p* = 0.015) were found to be protective alleles (Table 4). In addition, HLA-Cw6 was found to be positively associated with cutaneous psoriasis (OR = 2.50, *p* = 0.036) in the PsA subgroup (data not shown).

Alleles directly associated with uSpA were HLA-B16(38,39) (*p* = 0.02) and B27 (*p* < 0.001), whereas the protective alleles were Cw7 (*p* = 0.034) and DQ5 (*p* = 0.04), the latter not being recorded in any uSpA. In the ASAS^−^ subgroup, a direct association was found with HLA-A28(68) (*p* = 0.002), B15(62) (*p* = 0.049), Cw8 (*p* = 0.006), and Cw12 (*p* = 0.001) (Table 4).

With the only exception of HLA-DQ5 (not recorded in any uSpA), all statistically significant alleles in Fisher’s exact test were then subjected to multivariate (logistic regression) and multivariable analyses (Table 5).

In the former analysis, in which each single allele was analyzed using “gender” as confounding variable (Table 5), besides HLA-B27 (*p* = 0.003), the wE/A group showed a significant direct association with HLA-A28(68) (*p* = 0.005), Cw3 (*p* = 0.022), Cw8 (*p* = 0.018), and DQ1 (*p* = 0.003), while Cw7 (*p* = 0.014) and DR3 (*p* = 0.044) were confirmed to be protective.

In the PsA subgroup, alleles other than HLA-B27 (*p* = 0.002) showing a significant direct association were HLA-B17(57,58) (*p* = 0.017), DQ1 (*p* = 0.024), and DQ3 (*p* = 0.036), while Cw7 (*p* = 0.037) and DQ7 (*p* = 0.011) were protective, as found with the previous Fisher’s exact test.

The uSpA subgroup showed a significant association with HLA-B16(38,39) (*p* = 0.025) and B27 (*p* < 0.001) and the ASAS^−^ subgroup with HLA-A28(68) (*p* = 0.005) and Cw8 (*p* = 0.004).

At multivariable logistic regression analyses, the wE/A group showed significant independent associations with HLA-A28(68) (*p* = 0.047), B27 (*p* = 0.026), Cw3 (*p* = 0.004) and DQ1 (*p* = 0.008) (Table 5). When the same type of analysis was applied to E/A subgroups, the results showed that PsA was associated with HLA-B27 (*p* = 0.035) and DQ1 (*p* = 0.042), uSpA with HLA-B16(38,39) (*p* = 0.003) and B27 (*p* < 0.001), and E/A ASAS^−^ with HLA-28(68) (*p* = 0.006), and Cw8 (*p* = 0.006).

## 5. Discussion

To the best of our knowledge, this is the first study to investigate the association between HLA class I and class II alleles and E/A ASAS^−^(vs. ASAS^+^ PsA and uSpA) in a cohort of Caucasian patients (*n* = 113) as compared to an HLA-typed HD cohort (*n* = 318).

While previous studies investigated the association between PsA [22] and HLA-B27 only, our analysis was focused on alleles other than HLA-B27 in E/A patients and its subgroups, PsA, uSpA, and ASAS^−^.

In addition to HLA-B27, in the wE/A cohort, our data revealed a significant association with HLA-A28(68), Cw3, Cw12, and DQ1. All these associations, except the one with HLA-Cw12, were independent of HLA-B27, as demonstrated by multivariable regression analysis. Regarding HLA-Cw12, it was not possible to establish the interdependence with HLA-B27, because this allele was not present in any HD.

Interestingly, we found that both HLA-A28(68) and Cw12 were also associated with ASAS^−^ but not with PsA or uSpA, suggesting that these alleles can confer susceptibility to ASAS^−^. While HLA-A28, with its splits 68 and 69, has been demonstrated to be significantly associated with B27 risk-related diseases, including AS [23], reactive arthritis [23], juvenile chronic polyarthropathy [23], and intermediate uveitis [24], only a minimal predisposition to cutaneous psoriasis has been reported for HLA-Cw12 [25,26].

ASAS^−^, which was the only E/A subgroup not associated with HLA-B27, presented an additional association with HLA-Cw8, never previously found to be associated with E/A or its subgroups.

Regarding HLA-Cw3 and HLA-DQ1, both of which favor wE/A, the former was reported to have a statistically high prevalence in other immune-mediated diseases such as rheumatoid arthritis-associated vasculitis [27,28], while HLA-DQ1 was shown to be a predisposing factor for autoimmune uveitis, the most common extra-articular manifestation of SpA, in a cohort of Caucasian Italian patients [29]. Furthermore, HLA-DQ1 and HLA-DQ2 have been associated with the presence of anti-Ro/SSA antibodies in both Sjögren’s syndrome [30] and systemic lupus erythematosus patients [31].

In our study population, HLA-DQ1, along with HLA-B27, was also associated with PsA.

The prevalence of PsA patients positive for HLA-B27 in our investigation was 13%, comparable to the 12% reported by Paladini et al. [22] in an Italian Caucasian cohort.

Several studies have shown that HLA-Cw6 is strongly associated with cutaneous psoriasis, but not with PsA [32,33]. The lack of an established diagnosis of cutaneous psoriasis in 51% of our PsA group can explain the absence of this subgroup association with HLA-Cw6.

Another finding of this study is the association of HLA-B16 (38,39) with uSpA, although earlier reports suggested an HLA-B39 association with PsA [34,35], HLA-B27-negative AS [36,37], and pauci-articular juvenile chronic arthritis [37,38].

A major study limitation is the lack of longitudinal observations that could establish whether ASAS^−^ forms could eventually evolve into any other E/A subgroup, although the peculiar association of ASAS^−^ with HLA-Cw8 makes this possibility unlikely.

The role of gender in influencing some HLA associations deserves some comment in that certain HLA allotypes, found to be significantly associated with wE/A (HLA-DQ3), uSpA (HLA-Cw7) or ASAS^−^ (HLA-B15(62)) by Fisher’s exact test, lost their significant association when “gender” was included as covariate in the multivariate analyses. These findings suggest that gender had an impact on the expression of certain HLA alleles in the E/A groups in our study, in agreement with previous studies showing gender-related HLA differences in different autoimmune diseases [39,40,41,42].

Finally, our work paves the way for further investigation aimed at genotyping the HLA alleles found in this study to be associated with uSpA or ASAS^−^.

## 6. Conclusions

Our study revealed that HLA-B27 and HLA-B16(38,39) are significantly more frequent in uSpA patients, while HLA-A28(68), HLA-Cw8, and HLA-Cw12 were found to be associated with ASAS^−^ in the general population in Italy. These alleles can thus be associated with a diagnosis of ASAS^−^, and HLA typing may contribute to correct clinical management, as well as to identifying family members at risk, in particular those patients with enthesitis and/or arthritis who do not meet the criteria for a uSpA diagnosis. These findings warrant confirmation in further large, multicenter cohort studies.

## Figures and Tables

**Table 1:** Clinical characteristics of the 113 patients with peripheral seronegative enthesitis/arthritis (E/A), in the whole cohort, and the ASAS^+^ and ASAS^−^ subgroups.

Variable	Whole Cohort *n* = 113	ASAS^+^		ASAS^−^ *n* = 40
		PsA *n* = 54	uSpA *n* = 19	
Female (*n*; %)	92; 81.4	42; 79.2	19; 84.2	34; 85.0
Age (mean ± SD; median)	53.8 ± 11.4; 54	53.2 ± 9.5; 54.5	50.8 ± 15.2; 55	56.2 ± 11.8; 54
Age at the onset of first symptoms (mean ± SD; median)	44.5 ± 12.7; 44.6	43.4 ± 12.0; 42.15	40.8 ± 14.1; 43.2	47.6 ± 12.6; 46.9
Weight (mean ± SD; median)	72.4 ± 14.4; 70	72.9 ± 15.1; 70	70.7 ± 15.0; 75	71.7 ± 12.5; 70
High (mean ± SD; median)	164.1 ± 8.4; 164	165.1 ± 7.6; 165	163.7 ± 7.5; 165	162.8 ± 9.5; 165
BMI (mean ± SD; median)	26.7 ± 4.4; 26	26.7 ± 4.8; 26	26.2 ± 4.7; 26	26.9 ± 3.7; 27
BMI > 29.9 (*n*; %)	29; 25.6	12.0; 22. 6	5; 26.3	11.0; 27.5
Familiarity for psoriasis (*n*; %)	45; 39.8	39; 73.6	3; 18.79	3.0; 7.5
Cutaneous psoriasis (*n*; %)	26; 23.0	26; 49.1	0	0
Peripheral arthritis only (*n*, %)	103; 91.2	47; 88.7	16; 84.2	39; 97.5
Peripheral and axial arthritis (*n*; %)	10; 8.8	6; 11.3	3; 15.78	1; 2.5
Smoking (*n*; %)	26; 23.0	8; 15.1	7; 26.8	10; 25
Hypertension (*n*; %)	46; 40.7	26; 49.1	4; 21	15; 37.5
Cardiovascular risk (*n*; %) ^a^	37; 32.7	20; 37.7	5; 26.3	11; 27.5
Metabolic syndrome ^b^ (*n*; %)	5; 4.4	2; 3.8	2; 10.5	1; 2.5
Autoimmune thyroiditis (*n*; %)	16; 14.1	10; 18.9	2; 10.5	4;10
Fibromyalgia (*n*; %)	24; 21.2	12; 22.6	4; 21.0	8; 20
Malignancy (*n*; %)	10; 8.8	6; 11.3	1; 11.1	3; 7.5

BMI, body mass index; ASAS^−^, E/A patients responding to DMARDs, but not fulfilling ASAS criteria; PsA, psoriatic arthritis; uSpA, patients fulfilling ASAS classification criteria but not satisfying any of the classification criteria related to SpA subgroups. ^a^ Categorized as positive in the presence of any of the followings: diabetes, obesity, hypercholesterolemia, and metabolic syndrome. ^b^ Defined by the National Cholesterol Education Program (NCEP) Adult Treatment Panel III (ATP III) criteria.

**Table 2:** Fisher’s exact test to define clinical characteristics showing a statistically higher (odds ratio; OR > 1) or lower (OR < 1) prevalence in peripheral seronegative enthesitis/arthritis (E/A) ASAS^+^ (PsA and uSpA) vs. ASAS^−^ clinical subgroups.

Variable	PsA vs. uSpA	PsA vs. ASAS^−^	uSpA vs. ASAS^−^
	*p*; OR ^a^		
Female	0.745; 0.6	0.607; 0.7	1; 1.06
Age	0.355	0.329	0.221
Age at the onset of first symptoms	0.580	**0.03**	0.09
Weight	0.554	0.880	0.662
Height	0.465	0.058	0.425
BMI	0.811	0.671	0.685
BMI > 29.9	1; 0.88	0.817; 0.89	1; 1.06
Familiarity for psoriasis	<0.001; 13.86	<0.001; 33.8	0.072; 0.83
Cutaneous psoriasis	<0.001; NA	<0.001; NA	NA
Peripheral and axial arthritis	0.69; 0.66	0.132; 5.12	0.094; 0.13
Smoking	0.098; 0.31	0.216; 0.52	0.366; 0.56
Hypertension	0.033; 3.75	0.214; 1.80	0.247; 2.25
Cardiovascular risk	0.41; 1.78	0.275; 1.79	1; 1.06
Metabolic syndrome	0.276; 0.32	1; 1.57	0.240; 0.21
Autoimmune thyroiditis	0.72; 1.93	0.256; 2.15	1; 0.94
Fibromyalgia	1; 1.07	0.80; 1.21	1; 0.93
Malignancy	0.67; 2.25	0.727; 1.62	1; 1.45

ASAS^−^, patients responding to DMARDs, but not fulfilling ASAS criteria; BMI, Body Mass Index; NA, not applicable; OR, odds ratio; PsA, psoriatic arthritis; uSpA, patients fulfilling ASAS classification criteria but not satisfying any of the classification criteria related to SpA subgroups; ^a^ significance was assessed by Mann–Whitney *U* test for continuous variables (*p*), and Fisher’s exact test for nominal variables (*p*; OR). A *p* < 0.05 was considered statistically significant.

**Table 3:** HLA-B27 percentage in patients with peripheral seronegative enthesitis/arthritis (E/A) in the whole cohort (wE/A), in ASAS^+^ psoriatic arthritis (PsA) and uSpA, and in ASAS^−^ patients.

Group (*n* of Patients)	B27^+^ (*n*; %)	*p* ^a^
wE/A (113)	12; 10.61	0.001
PsA (54)	7; 13	0.001
uSpA (19)	5; 26.3	<0.001
ASAS^−^ (40)	0; 0	1
Healthy donors (318)	7; 2.2	NA

ASAS^−^, patients responding to DMARDs, but not fulfilling ASAS criteria; NA, not applicable; uSpA, patients fulfilling ASAS classification criteria but not satisfying any of the classification criteria related to SpA subgroups. ^a^ Fisher’s exact test for each group vs. healthy donors; *p* < 0.05 was considered statistically significant.

**Table 4:** Fisher’s exact test to define HLA allotypes showing a statistically higher (odds ratio; OR > 1) or lower (OR < 1) prevalence in peripheral seronegative enthesitis/arthritis (E/A) patients than in healthy donors (HD).

E/A Patients Grouping	Allotype	*p* ^a^	OR (95% CI)
Whole	A28(68)	0.001	11.12 (2.32–53.22)
	B27	0.001	5.27 (2.02–13.77)
	Cw3	0.022	2.29 (1.17–4.47)
	Cw7	0.004	0.50 (0.31–0.79)
	Cw8	0.023	4.44 (1.23–16.04)
	Cw12 ^b^	0.004	N/A
	DR3	0.008	0.16(0.03–0.71)
	DQ1	0.005	2.39 (1.32–4.31)
	DQ3	0.038	2.67 (1.09–6.26)
PsA	B17(57,58)	0.035	2.19 (1.05–4.33)
	B27	0.001	6.76 (2.22–19.71)
	Cw7	0.016	0.45 (0.25–0.90)
	DQ1	0.036	2.23 (1.08–4.61)
	DQ3	0.030	3.14 (1.15–8.54)
	DQ7	0.015	0.40 (0.20–0.82)
uSpA	B16(38,39)	0.02	3.61 (1.29–10.10)
	B27	<0.001	15.86 (4.47–56.29)
	Cw7	0.034	0.30 (0.09–0.94)
	DQ5 ^c^	0.04	NA
ASAS^−^	A28(68)	0.002	16.22 (2.86–91.71)
	B15(62)	0.049	5.07(1.16–22.10)
	Cw8	0.006	8.97 (2.14–37.46)
	Cw12	0.001	NA

ASAS^−^, patients responding to DMARDs, but not fulfilling ASAS criteria, CI, confidence interval; NA, not applicable; NI, not included; OR, odds ratio; PsA, psoriatic arthritis; uSpA, patients fulfilling ASAS classification criteria but not satisfying any of the classification criteria related to SpA subgroups; ^a^ Fisher’s exact test; a *p* < 0.05 was considered statistically significant. ^b^ Not recorded in any HD. ^c^ Not recorded in any uSpA.

**Table 5:** Logistic regression analyses to assess the interdependency of variables found to be statistically associated with peripheral seronegative enthesitis/arthritis (E/A) in the whole cohort or clinical subgroups (odds ratio; OR > 1), as compared to healthy donors (HD).

Disease and Subgroup	Allotype	Multivariate ^a^		Multivariable ^b^	
		OR (95% CI)	*p*	OR (95% CI)	*p*
Whole E/A	A28(68)	10.26 (2.02–51.94)	0.005	10.17 (1.03–100.02)	0.047
	B27	4.58 (1.69–12.44)	0.003	12.20 (1.34–111.00)	0.026
	Cw3	2.26 (1.25–4.56)	0.022	11.21 (2.18–57.6)	0.004
	Cw7	0.55 (0.34–0.88)	0.014	0.95 (0.44–1.84)	0.784
	Cw8	5.18 (1.32–20.32)	0.018	4.48 (0.33–59.81)	0.256
	Cw12 ^c^	NA	0.999	NA	0.999
	DR3	0.20 (0.04–0.95)	0.044	0	0.999
	DQ1	2.66 (0.94–5.92)	0.003	2.68 (1.29–5.55)	0.008
	DQ3	2.33 (0.94–5.92)	0.066	3.27 (1.01–10.61)	0.048
PsA	B17(57,58)	2.43 (1.17–5.07)	0.017	2.59 (0.87–7.67)	0.085
	B27	6.09 (1.96–18.92)	0.002	14.99 (1.29–186.25)	0.035
	Cw7	0.51 (0.27–0.96)	0.037	0.83 (0.36–1.91)	0.669
	DQ1	2.41 (1.12–5.17)	0.024	2.37 (1.03–5.46)	0.042
	DQ3	3.05 (1.07–8.68)	0.036	2.12 (0.60–7.52)	0.244
	DQ7	0.38 (0.18–0.80)	0.011	0.50 (0.21–1.16)	0.110
uSpA	B16(38,39)	3.81 (1.16–9.44)	0.025	5.63 (1.78–17.81)	0.003
	B27	13.56 (3.65–50.31)	<0.001	20.96 (5.08–86.36)	<0.001
	Cw7	0.34(0.10–1.05)	0.062	0.31 (0.09–1.04)	0.059
ASAS^−^	A28(68)	13.48 (2.19–82.68)	0.005	14.72 (2.18–99.28)	0.006
	B15(62)	4.27 (0.92–19.77)	0.063	7.54 (1.46–38.78)	0.016
	Cw8	9.96 (2.12–46.80)	0.004	9.97 (1.90–52.24)	0.006
	Cw12	NA	0.999	NA	0.999

ASAS^−^, E/A patients responding to DMARDs, but not fulfilling ASAS criteria; CI, confidence interval; NA, not applicable; NI, not included; OR, odds ratio; PsA, psoriatic arthritis; uSpA, patients fulfilling ASAS classification criteria but not satisfying any of the classification criteria related to SpA subgroups; ^a^ Alleles found statistically associated with E/A in Fisher’s exact test were analyzed by multivariate logistic regression, in which each single variable, tested for E/A vs. HD, was adjusted for gender. ^b^ Variables found statistically associated with E/A in Fisher’s exact test were analyzed by multivariable logistic regression. ^c^ Not recorded in any HD.

## Data Availability

The data presented in this study are available on request from the corresponding author. The data are not publicly available due to privacy restrictions.
